# Multi-omics analysis reveals changes in tryptophan and cholesterol metabolism before and after sexual maturation in captive macaques

**DOI:** 10.1186/s12864-023-09404-3

**Published:** 2023-06-07

**Authors:** Xu Liu, Xuyuan Liu, Xinqi Wang, Ke Shang, Jiawei Li, Yue Lan, Jiao Wang, Jing Li, Bisong Yue, Miao He, Zhenxin Fan

**Affiliations:** 1 Key Laboratory of Bioresources and Ecoenvironment (Ministry of Education), College of Life Sciences, Sichuan University, Chengdu, 610065 China; 2 Sichuan Key Laboratory of Conservation Biology on Endangered Wildlife, College of Life Sciences, Sichuan University, Chengdu, 610065 China; 3 Institute of Blood Transfusion, Chinese Academy of Medical Sciences, Chengdu, Sichuan China

**Keywords:** Rhesus macaques, Sexual maturity, Multi-omics, Gut microbiome, Blood transcriptome and metabolome

## Abstract

Rhesus macaques (*Macaca mulatta*, RMs) are widely used in sexual maturation studies due to their high genetic and physiological similarity to humans. However, judging sexual maturity in captive RMs based on blood physiological indicators, female menstruation, and male ejaculation behavior can be inaccurate. Here, we explored changes in RMs before and after sexual maturation based on multi-omics analysis and identified markers for determining sexual maturity. We found that differentially expressed microbiota, metabolites, and genes before and after sexual maturation showed many potential correlations. Specifically, genes involved in spermatogenesis (*TSSK2*, *HSP90AA1*, S*OX5*, *SPAG16*, and *SPATC1*) were up-regulated in male macaques, and significant changes in gene (*CD36*), metabolites (cholesterol, 7-ketolithocholic acid, and 12-ketolithocholic acid), and microbiota (*Lactobacillus*) related to cholesterol metabolism were also found, suggesting the sexually mature males have stronger sperm fertility and cholesterol metabolism compared to sexually immature males. In female macaques, most differences before and after sexual maturity were related to tryptophan metabolism, including changes in *IDO1*, *IDO2*, *IFNGR2*, *IL1Β*, *IL10*, L-tryptophan, kynurenic acid (KA), indole-3-acetic acid (IAA), indoleacetaldehyde, and *Bifidobacteria*, indicating that sexually mature females exhibit stronger neuromodulation and intestinal immunity than sexually immature females. Cholesterol metabolism-related changes (*CD36*, 7-ketolithocholic acid, 12-ketolithocholic acid) were also observed in female and male macaques. Exploring differences before and after sexual maturation through multi-omics, we identified potential biomarkers of sexual maturity in RMs, including *Lactobacillus* (for males) and *Bifidobacterium* (for females) valuable for RM breeding and sexual maturation research.

**Supplementary Information:**

The online version contains supplementary material available at 10.1186/s12864-023-09404-3.

## Introduction

Reaching sexual maturity is an important process in life during which sex organs and gonads develop rapidly, leading to the acquisition of fertility [1]. Exploring the occurrence, processes, and judgment criteria of animal sexual maturity has important scientific and economic value for understanding physiological changes in species and guiding their reproduction. Various studies on sexual maturation have been conducted across a wide range of species, such as RM [2], Atlantic salmon (*Salmo salar*) [3], Atlantic bluefin tuna (*Thunnus thynnus*) [4], Carib grackle (*Quiscalus lugubris*) [5], macaroni penguin (*Eudyptes chrysolophus*) [6], northern fur seal (*Callorhinus ursinus*) [7], and wild boar (*Sus scrofa*) [8].

As a model animal with a genetic background, phylogeny, and physiology similar to humans, RMs have made great contributions to the study of human physiology, disease, and behavior [9]. With the ever-increasing demand for captive RMs, how to efficiently guide breeding based on accurate sexual maturity criteria has become a key issue. Studies in captivity have suggested that sexual maturity in male macaques should be judged by sperm in semen samples, while that of females should be judged by the occurrence of at least two consecutive menstrual cycles [10, 11]. However, these judgments can be inaccurate and non-controllable, as spermatorrhea in males and menstruation in females may not always be readily detectable. Blood samples for sex hormone analysis can also be used to ascertain sexual maturity [12], but frequent sampling increases the complexity of the procedure. Several alternative parameters for determining sexual maturation have also been proposed, including age, weight, and testicular volume [10], but cannot reliably predict sexual maturity in non-human primates due to large inter-individual variability. Therefore, it is necessary to explore simpler and more accurate methods for judging sexual maturity in macaques.

With the development of high-throughput sequencing technology, multi-omics data contribute to explore biological processes from a variety of perspectives [13], including genomics [14], transcriptomics [15], metabolomics [16], microbiomics [17], and proteomics [18]. The effects of drugs on macaques have been explored using multi-omics analysis, greatly promoting biomedical research. For instance, multi-omics studies have shown that the antimalarial drug pyrimethamine inhibits cell division and metabolism of the RM immune system, affecting immune physiology [19]. Xu et al. found significant changes in tryptophan-related genes, metabolites and gut microbiomes in older RMs through the combined analysis of transcriptome, metabolome and metagenome [20]. Multi-omics can also effectively compare differences between multiple study individuals to reveal the impact of biologically relevant substances on life and the mechanisms underlying life processes. By comparing the blood transcriptome data of male and female RM infants, Yue et al. found 3 differentially expressed genes on the X chromosome and 14 differentially expressed genes on the Y chromosome [21]. Furthermore, blood transcriptome analysis has shown the similarities and differences in gene expression patterns between Tibetan macaques and humans, providing new insights into primate evolution [22]. Proteomic and transcriptomic analyses have revealed the role of fatty acid binding protein 4 in the pathogenesis of diet-induced diabetes in macaques [23]. Metatranscriptomic analysis of feces from macaques with idiopathic chronic diarrhea has helped clarify the disease process, implicating mucin degradation and changes in fucose utilization [24]. Besides, multi-omics has been widely used in studies of human sexual maturity. Aksnes et al. detected vitamin D-related metabolites in human blood and found that the concentration of vitamin D in blood during puberty was strongly correlated with sexual maturity [25]. Almstrup et al. analyzed metabolome and methylation data and found that the level of metabolite endocrine disrupting chemicals (EDC) is related to the DNA methylation profile of blood before and after puberty, indicating that environmental chemicals can potentially alter adolescent development [26]. However, there is substantial variation in physiological changes between individuals (within and between sexes) of the same (and different) species with regard to sexual maturation. Here, we aim to identify reliable markers to help judge sexual maturity in RMs by analyzing changes (differential microbiota, metabolites, and genes) before and after sexual maturity with a multi-omics approach. This study provides new insights into the judgment and mechanisms of sexual maturity in captive RMs, which may facilitate breeding and reproduction. The strategy and results from our study could also be applied to wild populations and even other protected animals.

## Materials and methods

### Animal management

In total, 28 macaques were divided into four groups: six sexually immature male macaques without ejaculation behavior (MNS; 1.5 years old); nine sexually mature male macaques with breeding experience (MS; 5–7 years old); seven sexually immature female macaques without menstruation behavior (FNS; 1.5 years old); six sexually mature female macaques with breeding experience (FS; 5–7 years old). All RMs were captive individuals from Sichuan Green-House Biotech Co., Ltd. (Meishan, Sichuan, China) and housed under the same conditions with peers in intact social group, all individuals were free to eat and drink during the experiment. All sample information and composition of feed are shown in Supplementary Table 1.

Our study protocol was approved by the Ethics Committee of College of Life Sciences, Sichuan University (No. 20200327012 and No. 20210308001). The guidelines of the Sichuan Experimental Animal Management Committee were strictly followed during sample collection.

### Sample collection

To obtain blood samples and fresh fecal samples from each individual, RMs from each group were housed in a single cage one day before sampling. Total 23 blood samples were collected using PAXgene Blood RNA tubes and 24 plasma samples were collected using heparin anticoagulant tubes without anesthesia, both blood and plasma samples were 2 ml. All sampling vessel should be gently reversed for 6–8 times before being temporarily placed in an ice box. Fecal samples were collected as soon as possible after animal defecation, after removing surface pollutants, 21 fecal samples were placed in sterile centrifuge tubes. Disposable gloves need to be replaced for the next sample. All blood and fecal samples were stored at − 80 °C.

### RNA sequencing and Differentially Expressed Gene (DEG) analysis

RNA samples were sent to Novogene (Beijing, China) for paired-end sequencing using the Illumina NovaSeq 6000 platform and 58,230,463.9 average raw reads were obtained. After quality control and read mapping, 57,954,233.86 final high quality reads was obtained by using NGS QC Toolkit v2.3.3 [27] and HISAT2 v2.1.0 [28], respectively. The DESeq2 R package [29] was used for DEG screening, and genes were considered as DEGs with *p* < 0.05 and log2 fold-change > 1. Functional enrichment analysis based on Gene Ontology (GO) and Kyoto Encyclopedia of Genes and Genomes (KEGG) [30] [31, 32] was performed using g:Profiler (http://biit.cs.ut.ee/gprofiler/gost) [33]. Specific analysis process is shown in Supplementary material 4.

### Metabolome database analysis

Plasma samples were used for metabolome. After thawing until no ice was present, the samples (50 μL) were vortexed for 3 min with 300 μL of pure methanol and centrifuged for 10 min at 12 000 rpm and 4℃. The supernatant (200 μL) was collected and set at − 20℃ of freezer for 30 min, then centrifuged for 3 min at 12 000 rpm and 4℃, with 150 μL of the resulting supernatant used for Liquid Chromatography-Tandem Mass Spectrometry (LC–MS) analysis. Ultra-performance liquid chromatography (UPLC) (ExionLC AD, https://sciex.com.cn/) and quadrupole time-of-flight mass spectrometry (TripleTOF 6600, AB SCIEX) were used for the LC-QTOF-MS/MS experiments. Accurate qualitative and quantitative analyses were performed using the self-established target standard database MWDB and integrated public database MHK. Differentially expressed metabolites (DEMs) were screened using univariate and multidimensional analysis. The *p*-values obtained from two tailed unpaired t-test and variable influence on projection (VIP) values obtained from the partial least squares discriminant analysis (PLS-DA) model were the main factors, *p* < 0.05 and VIP > 1 were used as the screening criteria for DEMs. Specific analysis process is shown in Supplementary material 4.

### Metagenomic sequencing and analysis

Extracted DNA samples were sent to Novogene (Beijing, China) for paired-end sequencing on the Illumina NovaSeq 6000 platform. After removing adapters, low-quality raw reads, and host pollution, we performed gene prediction and non-redundant construction using Prodigal [34] and CD-HIT [35]. Kraken2 [36] and linear discriminant analysis effect size (LEfSe) [37] were used for species annotation and screening of crucial species. DIAMOND [38] was used to build a dbCAN database and carbohydrate-active enzymes (CAZy) annotation [39]. Functional composition of species and quantification of gene families and pathways were performed using HUMANn3 [40]. QIIME2 was used to calculate alpha [41] and beta diversity [42]. Specific analysis process is shown in Supplementary material 4.

### Statistical analysis

The *p*-values obtained from two tailed unpaired t-test, *p* < 0.05 means a significant difference, and “ ∗ ” in figures represents *p* < 0.05. Correlation analysis was performed by using Spearman correlation analysis, and |correlation coefficient|> 0.8 represents a high correlation. The area under curve (AUC) was obtained from the receiver operating characteristic (ROC) curve by GraphPad Prism8. AUC value between 0.7 and 0.9 means the biomarker has certain diagnostic accuracy, and more than 0.9 represents the biomarker has high diagnostic accuracy.

## Results

### Host transcriptome

**Fig. 1:**
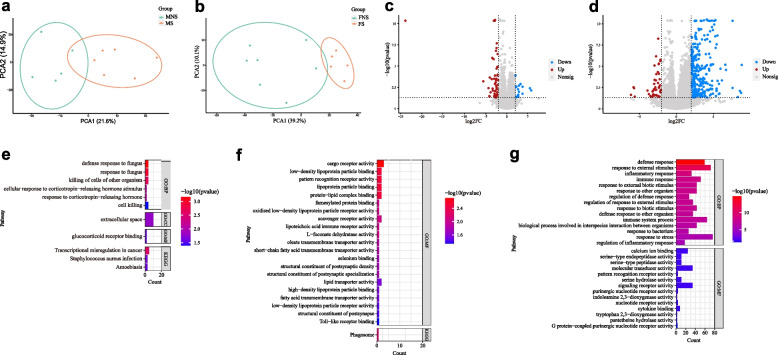
Blood transcriptome analysis. **a** Principal component analysis (PCA) between MS and MNS groups. **b** PCA between FS and FNS groups. **c** Volcano plots of DEGs between MS and MNS groups. **d** Volcano plots of DEGs between FS and FNS groups. **e** GO and KEGG pathway enrichment analyses of up-regulated DEGs in MS group (*p* < 0.05). **f** GO and KEGG pathway enrichment analyses of down-regulated DEGs in MS group (*p* < 0.05). **g** GO and KEGG pathway enrichment analyses of down-regulated DEGs in FS group (*p* < 0.05). A *p*-value cutoff of 0.05 as used as the level of significance

We performed transcriptome analysis of 23 macaques (11 males and 12 females). In total, 23,438 known genes were obtained after quality control, mapping, and removal of lowly expressed genes. Results showed no significant differences in gene expression between the MNS and MS groups (*p* > 0.05, Fig. 1a), but significant differences in gene expression between the FNS and FS groups (*p* < 0.05, Fig. 1b). In total, 291 genes were identified as DEGs between the MNS and MS groups (Fig. 1c) and 1,207 genes were identified as DEGs between the FNS and FS groups (false discovery rate (FDR) of 0.05) (Fig. 1d). In the MS group, 146 up-regulated DEGs were enriched in GO terms related to corticotropin-releasing hormone, such as glucocorticoid receptor binding (GO:0035259), response to corticotropin-releasing hormone (GO:0043435), and cellular response to corticotropin-releasing hormone stimulus (GO:0071376) (Fig. 1e), while 145 down-regulated DEGs were enriched in GO terms related to lipoprotein, such as low-density lipoprotein particle binding (GO:0030169), oxidized low-density lipoprotein particle receptor activity (GO:0150025), and low-density lipoprotein particle receptor activity (GO:0005041) (Fig. 1f). In the FS group, the 206 up-regulated DEGs were not enriched in any GO terms and KEGG pathways, while the 1 001 down-regulated DEGs were enriched in GO terms related to inflammation and tryptophan metabolism, such as inflammatory response (GO:0006954), tryptophan 2, 3-dioxygenase activity (GO:0004833), and indoleamine 2, 3-dioxygenase activity (GO:0033754) (Fig. 1g).

### Host metabolome

**Fig. 2:**
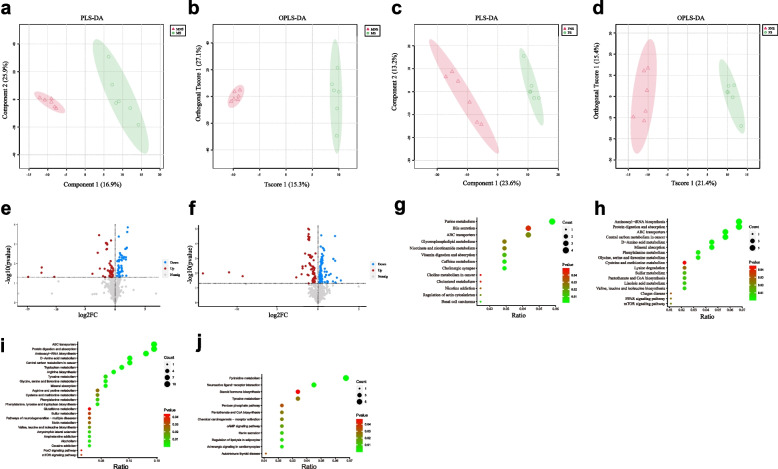
Blood metabolome analysis. **a** Partial least squares discriminant analysis (PLS-DA) score plots based on metabolic profiles between MS and MNS groups. **b** Orthogonal partial least squares discriminant analysis (OPLS-DA) score plots based on metabolic profiles between MS and MNS groups. **c** PLS-DA score plots based on metabolic profiles between FS and FNS groups. **d** OPLS-DA score plots based on metabolic profiles between FS and FNS groups. **e** Volcano plots of DEMs between MS and MNS groups (*p* < 0.05, VIP > 1). **f** Volcano plots of DEMs between FS and FNS groups (*p* < 0.05, VIP > 1). **g** Pathway enrichment analyses of up-regulated DEMs in MS group. **h** Pathway enrichment analyses of down-regulated DEMs in MS group. **i** Pathway enrichment analyses of up-regulated DEMs in FS group. **j** Pathway enrichment analyses of down-regulated DEMs in FS group

In total, 644 metabolites were identified in 24 RMs (12 males and 12 females) using the MetWare metabolite database (Chengdu, China). All raw metabolite data are shown in Supplementary Table 2. Of these metabolites, 96 were identified as significant DEMs between the MS and MNS groups, including 44 up-regulated and 52 down-regulated DEMs in the MS group (Fig. 2a, b, e). The up-regulated DEMs included hormones, hormone-related compounds, organic acid and derivatives, and carnitine, such as carnitine C13:1, carnitine C15:1, caffeic acid, cholesterol, and 8-isoprostaglandin F1α, which were enriched in cholesterol metabolism, bile secretion, and ABC transporters (Fig. 2g). The down-regulated DEMs included bile acids, oxidized lipids, and amino acids, such as orthocholic acid, 7-ketolithocholic acid, 13(R)-HODE, and L-arginine, which were enriched in D-amino acid metabolism, linoleic acid metabolism, mTOR signaling pathway, and PPAR signaling pathway (Fig. 2h).

In total, 158 metabolites were identified as significant DEMs between the FS and FNS groups, including 69 up-regulated and 89 down-regulated DEMs in the FS group (Fig. 2c, d, f). The main up-regulated DEMs in the FS group included amino acid derivatives, organic acid and its derivatives, and heterocyclic compounds, such as L-tryptophan, KA, IAA, and indoleacetaldehyde, which were enriched in tryptophan metabolism, mTOR signaling pathway, and neurodegeneration pathways (Fig. 2i). The main down-regulated DEMs in the FS group included hormone and lipid metabolism-related metabolites, such as estrone, epinephrine, cortisol, taurocholic acid sodium salt hydrate, and 7-ketolithocholic acid, which were enriched in steroid hormone biosynthesis, regulation of lipolysis in adipocytes, and adrenergic signaling in cardiomyocytes (Fig. 2j).

### Gut microbiota

**Fig. 3:**
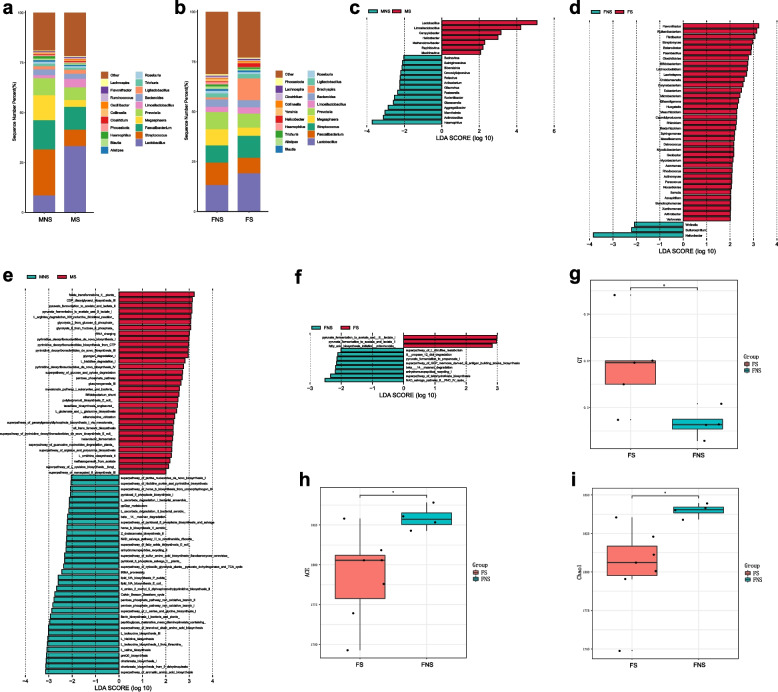
Metagenomic analysis of gut microbiota. **a** Top 20 abundant genera in gut between MS and MNS groups. **b** Top 20 abundant genera in gut between FS and FNS groups. **c** Differential analysis of gut microbial composition in MS and MNS groups. **d** Differential analysis of gut microbial composition in FS and FNS groups. **e** Differential analysis of gut microbial function in MS and MNS groups. **f** Differential analysis of gut microbial function in FS and FNS groups. **g** Differential analysis of gut microbial CAZy enzyme in FS and FNS groups. **h** Alpha diversity (ACE) estimates between FS and FNS groups. **i** Alpha diversity (Chao1) estimates between FS and FNS groups. A *p*-value cutoff of 0.05 as used as the level of significance

The gut microbiota detected in the four groups (21 RMs, 12 males and nine females) were classified into 82 phyla, 685 families, 2 632 genera, and 8 973 species. At the phylum level, Firmicutes, Bacteroidetes, and Proteobacteria were dominant in the MS, MNS, and FNS groups, while Firmicutes, Bacteroidetes, and Spirochaetes were dominant in the FS group. At the genus level, *Lactobacillus* (13.15%), *Faecalibacterium* (11.29%), and *Prevotella* (8.63%) were dominant in the FNS group; *Lactobacillus* (19.06%), *Streptococcus* (10.97%), and *Brachyspira* (10.94%) were dominant in the FS group; *Streptococcus* (23.09%), *Faecalibacterium* (14.64%), and *Megasphaera* (12.55%) were the dominant in the MNS group; and *Lactobacillus* (33.13%), *Faecalibacterium* (11.43%), and *Streptococcus* (8.27%) were dominant in the MS group (Fig. 3a-b).

The relative abundances of *Lactobacillus*, *Limosilactobacillus*, *Campylobacter*, and *Aviadenovirus* were higher in the MS group than in the MNS group (*p* < 0.05). The relative abundances of *Azospirillum*, *Rhodococcus*, *Bifidobacterium*, and *Arthrobacter* were higher in the FS group than in the FNS group ((*p* < 0.05, Fig. 3c-d). The microbial metabolic pathways showed that amino acid and lipid biosynthesis-related pathways were decreased in the MS group, including the superpathway of branched chain amino acid biosynthesis, superpathway of aromatic amino acid biosynthesis, and lipid IVA biosynthesis (*E. coli*) (*p* < 0.05) (Fig. 3e). In the FS group, the fatty acid biosynthesis and pyruvate fermentation pathways were increased, including the superpathway of GDP-mannose-derived O-antigen building blocks biosynthesis and pyruvate fermentation to acetate and (S)-lactate I (*p* < 0.05) (Fig. 3f). In addition, the glycosyl transferases (GTs) of the CAZy enzyme families were significantly up-regulated in the FS group (*p* < 0.05) (Fig. 3g). However, there was no significant change in the CAZy enzyme families between the MS and MNS groups (*p* > 0.05). The alpha-diversity results showed no significant differences in the ACE, Chao1, Shannon, and Simpson indices between the MS and MNS groups (*p* > 0.05), whereas the ACE and Chao1 indices were significantly lower in the FS group compared to the FNS group (*p* < 0.05) (Fig. 3h-i).

### Association analysis between multi-omics

In order to further explore the differences of macaques before and after sexual maturity at different levels, we performed the correlation analysis by spearman. Based on the results of the above multi-omics analysis, we focused on the association between the differential microbiota, differential metabolites and differential genes related to tryptophan and cholesterol. For female, the correlation between differential microbiota and differential metabolites showed that IAA and Indoleacetaldehyde were highly correlated with *Rhodococcus* (|Correlation Coefficient|> 0.8, *p* < 0.05); the correlation between differential metabolites and differential genes showed that L-tryptophan and KA were highly correlated with *IDO2*, *IFNGR2* (|Correlation Coefficient|> 0.8, *p* < 0.05). For male, the correlation between differential microbiota and differential metabolites showed that 12-ketolithocholic acid and 7-ketolithocholic acid were highly correlated with *Aggregatibacter* (|Correlation Coefficient|> 0.8, *p* < 0.05); the correlation between differential metabolites and differential genes showed that 12-ketolithocholic acid and 7-ketolithocholic acid were highly correlated with *HSP90AA1*, *TSSK2* and *SPAG16* (|Correlation Coefficient|> 0.8, *p* < 0.05, Supplementary Table 3).

**Fig. 4:**
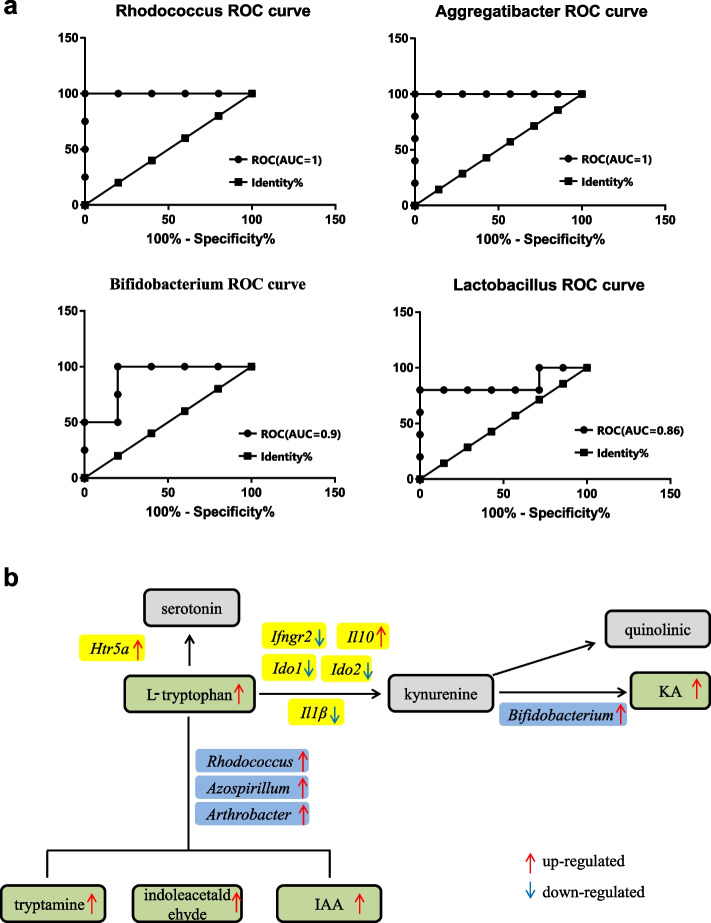
**a **ROC curve.** b** Three tryptophan metabolic pathways in FS group. 1. Tryptophan-serotonin pathway, elevation in plasma tryptophan and *Htr5a* expression in FS group indicated that the serotonin pathway was more active than that in the FNS group. 2. Tryptophan-kynurenine pathway, changes in *Ido1*, *Ido2*, *Ifngr2*, *Il1β*, and *Il10* indicated that the kynurenine pathway of tryptophan metabolism was inhibited in FS macaques. Increased KA indicated that the metabolism of kynurenine in the FS macaques was mainly along the KA pathway over the more neurotoxic quinolinic pathway. 3. Tryptophan-AhR ligand, tryptophan can be metabolized to AhR ligand (IAA) through gut microbiota. IAA: indole-3-acetic acid, KA: kynurenic acid

Moreover, combined with multi-omics difference analysis and correlation analysis, ROC curve analysis was performed for *Bifidobacterium*, *Rhodococcus*, *Lactobacillus* and *Aggregatibacter*. The result showed that the AUC value of *Bifidobacterium* (AUC = 0.9), *Rhodococcus* (AUC = 1), *Lactobacillus* (AUC = 0.86) and *Aggregatibacter* (AUC = 1) were all more than 0.8, suggesting that the *Bifidobacterium*, *Rhodococcus*, *Lactobacillus* and *Aggregatibacter* were all accurate as biomarkers (Fig. 4a).

## Discussion

In this study, we performed multi-omics analysis to characterize changes and identify potential biomarkers in RMs before and after sexual maturation. In the MS macaques, various spermiogenesis-related DEGs were identified, including *TSSK2*, *HSP90AA1*, S*OX5*, *SPAG16*, and *SPATC1*. Testis-specific serine/threonine kinase 2 (TSSK2), which is expressed exclusively in spermatids, is a member of the TSSK family and plays an essential role in male fertility [43]. Double knockout of *TSSK1*/*TSSK2* results in abnormal sperm development in mice [44]. Heat shock protein 90 (HSP90) is a key factor affecting post-meiotic differentiation of mammalian sperm [45, 46]. Targeted disruption of the *HSP90AA1* gene can lead to male infertility in mice [45]. Furthermore, HSP90 can prevent ubiquitination and degradation of TSSKs and is critical for their activation [46]. Sperm-associated antigen 16 L (SPAG16L), a major transcript isoform of *SPAG16*, encodes proteins related to cilia/flagella formation and motility [47]. SPAG16L deficiency can lead to male infertility, associated with impaired sperm motility [48]. Short SRY-box transcription factor 5 (S-SOX5), a form of *SOX5* transcript, is expressed in post-meiotic round spermatids [49, 50]. S-SOX5 may participate in the formation of motile cilia/flagella via activating SPAG16L [51]. Similarly, down-regulation of the spermatogenesis and centriole associated 1 (*SPATC1*) gene can reduce sperm fertility, although the exact function of *SPATC1* is unknown [52]. Thus, the up-regulation of these DGEs (*TSSK2*, *HSP90AA1*, S*OX5*, *SPAG16*, and *SPATC1*) in our study suggests that MS macaques have better sperm fertility, an important criterion of sexual maturity.

In the FS macaques, *MMP9*, a member of the matrix metalloproteinase (MMP) family, was down-regulated. MMPs (e.g., MMP1, MMP2, and MMP9) are involved in various physiological processes such as ovulation [53]. Luteinizing hormone (LH) and follicle stimulating hormone (FSH) are important factors in maintaining *MMP9* transcription in porcine granulosa cells [54]. However, LH and steroids can inhibit *MMP9* expression in RMs [55]. The decrease in MMPs suggests that LH and steroid levels may be increased in the FS group. Furthermore, we also found that estrone and epinephrine were significantly decreased in the FS group, similar to that reported in human studies [56]. Increased concentrations of free dehydroepiandrosterone and androstenedione, two sex steroids primarily derived from the adrenal glands [57], precede increases in gonadal-related hormones [58–60] during pubertal development. Before sexual maturity, estrogen may be converted peripherally through the adrenal hormones in adipose tissue with limited ovarian activity [60]. In our study, the levels of estrone, the primary estrogen in the peripheral conversion of epinephrine, were higher in the FNS group.

Using multi-omics analysis, we observed two striking differences in individuals before and after sexual maturity related to cholesterol and tryptophan metabolism. As a precursor of adrenocortical, estrogenic, and androgenic hormones, cholesterol is essential during sexual maturation [61]. In macaques, cholesterol is a recognized regulator of male fertility and cholesterol homeostasis is essential for sperm maturation [62]. Here, the expression of *CD36*, which is associated with cholesterol transport, was decreased in the MS group. Endogenous CD36 mediates the endocytosis of native low-density lipoproteins (LDLs) [63], which is required for cholesterol transfer [64]. High intracellular cholesterol can reduce cholesterol uptake by inhibiting the expression of LDL receptor genes [65]. Decreased *CD36* expression may lead to elevated plasma cholesterol, suggesting that male macaques have sufficient cholesterol for the synthesis of various steroids in sex organs after sexual maturity.

Consistent with the transcriptome results, plasma cholesterol levels were increased in the MS group. To maintain body homeostasis, excess cholesterol can be metabolized through the bile acid synthesis pathway. Cholesterol is crucial for the synthesis of primary bile acids, which generate secondary bile acids (7-ketolithocholic acid and 12-ketolithocholic acid) that are regulated by the microbiota [66]. Primary bile acids are converted into secondary bile acids by microbial deconjugation and 7α- dehydroxylation in the gut [67]. Microbial deconjugation involves the removal of glycine or taurine conjugates and is mainly performed by bacteria with bile salt hydrolase activity (BSH). Research has demonstrated that BSH exists in almost all major bacteria in the human gut, including *Lactobacillus*, *Bifidobacteria*, and *Clostridium* [66, 68, 69]. Microbial metabolism of cytotoxic bile acids facilitates their elimination through feces [67]. In our study, the abundance of BSH-expressing *Lactobacillus* increased, and plasma secondary bile acids decreased, suggesting that the MS macaques had better resistance to bile toxicity compared to the MNS macaques. Similarly, *CD36* levels were down-regulated in the FS macaques, although there were no significant differences in plasma cholesterol compared to the FNS group. In addition, BSH-expressing *Bifidobacteria* was increased in the FS macaques.

Our results also showed marked differences in tryptophan metabolism between the sexually mature and immature individuals (Fig. 4b). For female macaques, we found that tryptophan and KA, important metabolites of the tryptophan-kynurenine pathway, were significantly increased in the FS group. Most tryptophan (~ 90%) is metabolized through the kynurenine pathway [70]. KA is the downstream metabolite of the kynurenine pathway and plays a neuroprotective role in the central nervous system [71]. In our study, Several DEGs participate in the regulation of the tryptophan-kynurenine pathway, including *IDO1*, *IDO2*, *IFNGR2*, *IL1Β*, and *IL10*. Both indoleamine-2, 3-dioxygenase1 (IDO1) encoded by *IDO1* and indoleamine-2, 3-dioxygenase 2 (IDO2) encoded by *IDO2*, major rate-limiting enzymes of tryptophan metabolism [72], were decreased in the FS group. The down-regulated of *IFNGR2* encodes the interferon γ (IFN-γ) receptor and the down-regulated of *IL1Β* encodes interleukin-1 β (IL-1β), indicated the decreased of IFN-γ and IL-1β. IFN-γ and IL-1β have been identified as the important inducers of IDO1 [73, 74]. Furthermore, *IL10*, which encodes interleukin-10 (IL-10), a negative regulator of IDO1 [75], was increased in the FS group. These changes indicated that the tryptophan metabolism-related kynurenine pathway was inhibited in the FS macaques. However, there were no significant differences in kynurenine levels before and after sexual maturity, indicating that the metabolic rate of kynurenine was slower in the FS macaques. In investigating the potential antidepressant properties of *Bifidobacterium*, [76] found an increase in the concentrations of plasma tryptophan and KA without an increase in kynurenine in the *Bifidobacterium*-treated group, consistent with our results. Thus, the increased abundance of *Bifidobacterium* suggests that the FS macaques may possess more antidepressant compared to the FNS macaques. Interestingly, the two metabolic pathways of kynurenine are the KA pathway and quinolinic pathway, and the elevated KA levels suggested that the metabolism of kynurenine in the FS macaques was mainly along the KA pathway over the more neurotoxic quinolinic pathway. In contrast to the kynurenine pathway, the elevated levels of plasma tryptophan (serotonergic precursor) and *HTR5A* expression provide evidence that the serotonin pathway, which is related to adolescent mood control [77], was more active after sexual maturity in our study. Studies have shown that serotonin and dopamine are the major neurotransmitters involved in adolescent behavior, such as mood lability and high-risk behaviors [78, 79].

In addition to the kynurenine and serotonin pathways, tryptophan can also be metabolized through the gut microbiota. Tryptamine formed by tryptophan decarboxylation is oxidized to indoleacetaldehyde and converted to IAA. In our study, microbial tryptophan metabolites (tryptamine, IAA, and indoleacetaldehyde) were significantly increased in the FS group, suggesting tryptamine-IAA metabolic pathway activity. In addition to being a neuromodulator in the mammalian brain [80], tryptamine also acts as a signaling molecule to affect intestinal motility in the mammalian intestine [81–83]. Studies have shown that indole aldehyde, a short-lived metabolic intermediate, can influence intestinal immune responses via the aryl hydrocarbon receptor (AhR) [84]. IAA is known as a plant growth hormone. *Azospirillum* and *Arthrobacter* mainly metabolize tryptophan to IAA in plants [85, 86]. *Rhodococcus* regard as a crucial role in tryptophan degradation due to the upregulated of Indoleamine 2, 3-dioxygenase in *Rhodococcus* infected cells [87]. In mammals, as a metabolite of the gut microbiota and AhR ligand [88], IAA not only possesses the ability to scavenge free radicals but can also reduce the release of proinflammatory cytokines and regulate lipid metabolism [89–91]. Moreover, research on tryptophan metabolism in older RMs indicates that tryptophan is metabolized by microbiota to produce AhR ligands, ultimately affecting intestinal immunity [20]. Our results showed the female macaques possessed stronger emotional regulation, behavioral control, and intestinal immunity after sexual maturity. However, compared to female macaques, male macaques did not show marked differences in tryptophan metabolism after sexual maturity, similar to human studies showing higher rates of depression in females [92].

In summary, we applied multi-omics analyses (transcriptomics, metagenomics, and metabolomics) to study changes before and after sexual maturity in captive RMs and found many potential links between differentially expressed microbiota, metabolites, and genes involved in cholesterol and tryptophan metabolism. Notably, cholesterol metabolism-related changes before and after sexual maturity were found in both female and male macaques, while tryptophan metabolism-related changes before and after sexual maturity were mainly found in female macaques. In brief, the MS macaques exhibited stronger sperm fertility and cholesterol metabolism compared to the MNS macaques, and the FS macaques exhibited stronger neuromodulation and intestinal immunity compared to the FNS macaques. Furthermore, based on the results of multi-omics analyses and convenient sample collection, we suggest that *Lactobacillus* (for males) and *Bifidobacterium* (for females) could be used as potential markers of sexual maturity. Thus, we explored changes in RMs before and after sexual maturity and identified several potential biomarkers that may guide screening for mature RMs.

### Limitations of the study

There were two limitations to this study. Firstly, the sample size was not large enough, so the ROC analysis, which should based on the sufficient sample size, was not accurate enough. Next, age is one of the auxiliary criteria to judge sexual maturity and we did not exclude age as an interfering factor, but age may be the potential confounding variable in this study.

## Supplementary Information

**Additional file 1: Supplementary Table 1.** All samples information and composition of feed.

**Additional file 2: Supplementary Table 2.** Total 644 metabolites were identified.

**Additional file 3: Supplementary Table 3.** Association analysis.

**Additional file 4: Supplementary material 4.** The details of multi-omics analysis.

## Data Availability

All data is available in the manuscript or the supplementary materials. The raw data of transcriptomes and metagenomes have been submitted to the China National GeneBank DataBase (CNGBdb) with the accession number CNP0003577 (https://db.cngb.org/search/project/CNP0003577/).
