# Supplementary material for: Multi-omics analysis reveals changes in tryptophan and cholesterol metabolism before and after sexual maturation in captive macaques
Source: BMC Genomics. 2023 Jun 7;24:308. doi: 10.1186/s12864-023-09404-3 (PMC10249198; doi:10.1186/s12864-023-09404-3)
Supplement: Supplementary file 4 — Additional file 4: Supplementary material 4. The details of multi-omics analysis. [file 12864_2023_9404_MOESM4_ESM.docx]

**Materials and Methods**

**Library preparation and RNA sequencing**

A total amount of 3 μg RNA per sample was used as input material for the RNA sample preparations. Total RNA was extracted following the manufacturer’s PAXgene Blood RNA kit manual. RNA quality was assessed with an Agilent 2100 Bioanalyzer (Agilent Technologies, Santa Clara, CA). Total RNA was treated with the GlobinZero kit (Epicentre, Illumina, Madison, WI) and purified with a modified Qiagen RNeasy MinElute (Qiagen Inc., Valencia, CA) cleanup procedure or ethanol precipitation. Samples with RIN (RNA integrity number) values higher than 7.5 were used for library construction and sequencing. Library preparation and all sequencing runs were performed following manufacturer’s instructions. Single-stranded RNA was transformed into cDNA in a reverse transcript PCR approach using a strand-specific kit from Epicentre (ScriptSeq v2 Library Prep kit, Illumina, Madison, WI) with ScriptSeq Index PCR primers (Epicentre, Illumina, Madison, WI). After quantification with the KAPA SYBR Fast qPCR library quantification kit (Kapa Biosystems Inc.,Wilmington, MA), double-stranded cDNA was sequenced following the general methods descripted by (Fan, et al. 2014). Briefly, each cDNA sample was sonicated to fragments of 300~500bp in size and applied to paired-end libraries generation, then all of the libraries were sequenced using the Illumina Novaseq 6000 with a paired-end sequencing length of 150 bp (PE150) at Novogene (Beijing, China).

**Read** **alignment and quality control**

Rhesus macaque genome assembly Mmul_10 and reference annotation were download from Ensembl release-98 (www.ensembl.org). In order to obtain high quality reads, we employed NGS QC Toolkit v2.3.3 (Patel and Jain 2012) with stringent criteria (high-quality paired reads with more than 90% of bases with Q-value ≥ 20 were retained) to remove the low-quality paired-end reads or reads containing adaptors. Processed reads from each sample were mapped to the reference genome using HISAT2 v2.1.0 (Kim, et al. 2015). Each of the alignment output files were assembled into separate transcriptomes using StringTie v1.3.6 (Pertea, et al. 2015), which produces a transcript GTF file. Then, the Ensembl GTF file was used as the reference annotation file to guide the assembly process to get the expression value of TPM (transcripts per million) and raw read counts for each gene and transcripts.

**Gene clustering analysis and differentially expressed gene analysis**

Based on the expression values, PCA from the plotPCA of DESeq2 R package (Love, et al. 2014) was used to visualize the relatedness of 32 RNA-seq samples. We also used agglomerative hierarchical clustering in the ComplexHeatmap R package (Gu, et al. 2016) to perform clustering analysis. Differential expression analysis was also performed using DESeq2 that takes the raw read count as the input. The results of all statistical tests were corrected for multiple testing with the Benjamini-Hochberg false discovery rate (FDR ≤ 0.05) and an absolute value of log 2 fold change ≥ 2 was used to determine significant differences in gene expression.

**Gene enrichment analysis**

To functional classify the DEGs, we performed GO and KEGG enrichment analysis using g:Profiler (Raudvere, et al. 2019) and the background gene set was derived from the Ensembl annotation of macaque (e98_eg45_p14_cdb968b), consistent with the reference file we used for assembly. An FDR cut-off of 0.05 was considered to be significantly enriched.

**Metagenomic Analysis**

Total DNA from the swabs was extracted using a Tiangen DNA Stool Mini Kit (Tiangen Biotech Co., Ltd., China) and sent to Novogene (Beijing, China) for sequencing using the Illumina NovaSeq 6000 platform with a paired-end sequencing length of 150 bp. After sequencing, adapters and low-quality raw reads were removed using Trimmomatic based on a four-base wide sliding window, with average quality per base > 20 and minimum length 90 bp (Bolger et al., 2014). The CRM potential sequences were removed using Bowtie2 (Langmead and Salzberg, 2012) as part of the KneadData pipeline (https://github.com/biobakery/kneaddata) with the CRM reference genome (assembly Mmul_10). *De novo* assembly of the metagenomes from the quality-filtered Illumina reads was performed using MEGAHIT (Li et al., 2015) with the option “-t 96 –m 0.95 --min-contig-len 300”. Separate assembly of each sample was applied, rather than co-assembly of all samples, the advantages and disadvantages of which have been discussed previously (Pasolli et al., 2019). Gene prediction was performed using Prodigal (Hyatt et al., 2010) with the option “-p meta –g 11”. Non-redundant gene sets with thresholds of 95% similarity and 90% coverage of query sequences were constructed with CD-HIT (Fu et al., 2012) with the option “-c 0.95 -aS 0.90”. The non-redundant genes were further translated into amino acid sequences. The amino acid sequences were aligned using DIAMOND (Buchfink et al., 2015) with the option “--id 80% --query-cover 70% --evalue 1e-5” in the Carbohydrate-Active enZYmes (CAZy) database (Lombard et al., 2014). Quantification of the non-redundant genes in each metagenome was performed using Salmon (Patro, 2017) with the option “--meta”. Total abundance of each gene type was determined by total abundance of all genes mapped to the same gene type. Gene family and microbial metabolic pathway abundances were assessed using HUMANn3 (Franzosa, 2018) with the ChocoPhlAn and UniRef90 EC filtered databases (Suzek et al., 2007), and were normalized by copies per million (CPMs). Taxonomic labels of metagenomic sequences were assigned using Kraken2 (Wood and Salzberg, 2014) with the option “--use-mpa-style”. Taxon abundances were normalized by relative abundance. Differences in taxon, functional gene, and metabolic pathway abundances were determined using linear discriminant analysis effect size (LEfSe) (Segata et al., 2011). Antibiotic resistance genes (ARGs) were quantified using ShortBRED (Kaminski et al., 2015). In brief, the shortbred_identify.py script was used to produce a FASTA file of markers using the ARG sets in the Comprehensive Antibiotic Resistance Database (CARD) (Alcock et al., 2019) as proteins of interest and UniRef90 sequences as reference proteins, and the shortbred_quantify.py script was used to quantify the abundance of ARGs in the metagenomes. The ARG and gut microbiome abundance network was drawn using Cytoscape (Otasek et al., 2019).

**Diversity Analyses**

The taxonomic abundance table generated using Kraken2 was used as input for QIIME2 (Bolyen et al., 2019). The QIIME2 diversity plugin was used to calculate alpha (α) diversity (within sample diversity) (Bolyen et al., 2019). The QIIME2 plugin DEICODE (Martino et al., 2019) was used to calculate beta diversity with feature loadings.

**Metabolome Data Analysis**

Plasma samples were used for widely targeted metabolomics. After thawing until no ice was present, the samples (50 μL) were vortexed for 3 min with 300 μL of pure methanol and centrifuged for 10 min at 12 000 rpm and 4℃. The supernatant (200 μL) was collected and set at −20℃ for 30 min, then centrifuged for 3 min at 12 000 rpm and 4℃, with 150 μL of the resulting supernatant. The sample extracts were analyzed using an LC-ESI-MS/MS system (UPLC, ExionLC AD https://sciex.com.cn/; MS, QTRAP System, https://sciex.com/. The analytical conditions were as follows, UPLC: column, Waters ACQUITY UPLC HSS T3 C18 (1.8μm, 2.1 mm*100 mm); column temperature, 40°C; flow rate, 0.4mL/min; injection volume, 2μL or 5μL; solvent system, water (0.1% formic acid): acetonitrile (0.1% formic acid); gradient program, 95:5 V/V at 0 min, 10:90 V/V at 10.0 min, 10:90 V/V at 11.0 min, 95:5 V/V at 11.1min, 95:5 V/V at 14.0 min.

The effluent was alternatively connected to an ESI–triple quadrupole-linear ion trap (QTRAP)-MS. LIT and triple quadrupole (QQQ) scans were acquired on a triple quadrupole-linear ion trap mass spectrometer (QTRAP), QTRAP LC-MS/MS System, equipped with an ESI Turbo Ion-Spray interface, operating in positive and negative ion mode and controlled by Analyst 1.6.3 software (Sciex). The ESI source operation parameters were as follows: source temperature 500°C; ion spray voltage (IS) 5500 V (positive), -4500 V (negative);ion source gas I (GSI), gas II (GSII), curtain gas (CUR) were set at 50, 50, and 25.0 psi, respectively; the collision gas (CAD) was high. Instrument tuning and mass calibration were performed with 10 and 100 μmol/L polypropylene glycol solutions in QQQ and LIT modes, respectively. In the qualitative and quantitative analysis, the monitoring reaction (MRM) mode was used. The peak area of each chromatogrampeak represents the relative content of the correspondingmetabolites, MRM peak areas were measured by using Analyst 1.6.3. While accurate qualitative were performed using the self-established target standard database MWDB and integrated public database MHK.

**References**

Fan ZX, Zhao G, Li P, Osada N, Xing JC, Yi Y, Du LM, Silva P, Wang HX, Sakate R, et al. 2014. Whole-Genome Sequencing of Tibetan Macaque (Macaca thibetana) Provides New Insight into the Macaque Evolutionary History. Molecular Biology and Evolution 31:1475-1489.

Gu ZG, Eils R, Schlesner M. 2016. Complex heatmaps reveal patterns and correlations in multidimensional genomic data. Bioinformatics 32:2847-2849.

Kim D, Landmead B, Salzberg SL. 2015. HISAT: a fast spliced aligner with low memory requirements. Nature Methods 12:357-U121.

Love MI, Huber W, Anders S. 2014. Moderated estimation of fold change and dispersion for RNA-seq data with DESeq2. Genome Biology 15.

Patel RK, Jain M. 2012. NGS QC Toolkit: A Toolkit for Quality Control of Next Generation Sequencing Data. Plos One 7.

Pertea M, Pertea GM, Antonescu CM, Chang TC, Mendell JT, Salzberg SL. 2015. StringTie enables improved reconstruction of a transcriptome from RNA-seq reads. Nature Biotechnology 33:290-+.

Raudvere U, Kolberg L, Kuzmin I, Arak T, Adler P, Peterson H, Vilo J. 2019. g:Profiler: a web server for functional enrichment analysis and conversions of gene lists (2019 update). Nucleic Acids Research 47:W191-W198.

Bolger, A. M., Lohse, M., and Usadel, B. (2014). Trimmomatic: a flexible trimmer for Illumina sequence data. Bioinformatics 30, 2114–2120. doi: 10.1093/bioinformatics/btu170.

Langmead, B., and Salzberg, S. L. (2012). Fast gapped-read alignment with Bowtie 2. Nat Methods 9, 357–359. doi: 10.1038/nmeth.1923.

Li, D., Liu, C.-M., Luo, R., Sadakane, K., and Lam, T.-W. (2015). MEGAHIT: an ultra-fast single-node solution for large and complex metagenomics assembly via succinct de Bruijn graph. Bioinformatics 31, 1674–1676. doi: 10.1093/bioinformatics/btv033.

Pasolli, E., Asnicar, F., Manara, S., Zolfo, M., Karcher, N., Armanini, F., et al. (2019). Extensive Unexplored Human Microbiome Diversity Revealed by Over 150,000 Genomes from Metagenomes Spanning Age, Geography, and Lifestyle. Cell 176, 649-662.e20. doi: 10.1016/j.cell.2019.01.001.

Hyatt, D., Chen, G.-L., LoCascio, P. F., Land, M. L., Larimer, F. W., and Hauser, L. J. (2010). Prodigal: prokaryotic gene recognition and translation initiation site identification. BMC Bioinformatics 11, 119. doi: 10.1186/1471-2105-11-119.

Fu, L., Niu, B., Zhu, Z., Wu, S., and Li, W. (2012). CD-HIT: accelerated for clustering the next-generation sequencing data. Bioinformatics 28, 3150–3152. doi: 10.1093/bioinformatics/bts565.

Buchfink, B., Xie, C., and Huson, D. H. (2015). Fast and sensitive protein alignment using DIAMOND. Nat Methods 12, 59–60. doi: 10.1038/nmeth.3176.

Lombard, V., Golaconda Ramulu, H., Drula, E., Coutinho, P. M., and Henrissat, B. (2014). The carbohydrate-active enzymes database (CAZy) in 2013. Nucleic Acids Research 42, D490–D495. doi: 10.1093/nar/gkt1178.

Patro, R. (2017). Salmon provides fast and bias-aware quantification of transcript expression. Nature Methods, 10. doi: 10.1038/nmeth.4197.

Franzosa, E. A. (2018). Species-level functional profiling of metagenomes and metatranscriptomes. Nature MethOds 15, 12. doi: 10.1038/s41592-018-0176-y.

Suzek, B. E., Huang, H., McGarvey, P., Mazumder, R., and Wu, C. H. (2007). UniRef: comprehensive and non-redundant UniProt reference clusters. Bioinformatics 23, 1282–1288. doi: 10.1093/bioinformatics/btm098.

Wood, D. E., and Salzberg, S. L. (2014). Kraken: ultrafast metagenomic sequence classification using exact alignments. Genome Biol 15, R46. doi: 10.1186/gb-2014-15-3-r46.

Segata, N., Izard, J., Waldron, L., Gevers, D., Miropolsky, L., Garrett, W. S., et al. (2011). Metagenomic biomarker discovery and explanation. Genome Biol 12, R60. doi: 10.1186/gb-2011-12-6-r60.

Kaminski, J., Gibson, M. K., Franzosa, E. A., Segata, N., Dantas, G., and Huttenhower, C. (2015). High-Specificity Targeted Functional Profiling in Microbial Communities with ShortBRED. PLoS Comput Biol 11, e1004557. doi: 10.1371/journal.pcbi.1004557.

Alcock, B. P., Raphenya, A. R., Lau, T. T. Y., Tsang, K. K., Bouchard, M., Edalatmand, A., et al. (2019). CARD 2020: antibiotic resistome surveillance with the comprehensive antibiotic resistance database. Nucleic Acids Research, gkz935. doi: 10.1093/nar/gkz935.

Otasek, D., Morris, J. H., Bouças, J., Pico, A. R., and Demchak, B. (2019). Cytoscape Automation: empowering workflow-based network analysis. Genome Biol 20, 185. doi: 10.1186/s13059-019-1758-4.

Bolyen, E., Rideout, J. R., Dillon, M. R., Bokulich, N. A., Abnet, C. C., Al-Ghalith, G. A., et al. (2019). Reproducible, interactive, scalable and extensible microbiome data science using QIIME 2. Nat Biotechnol 37, 852–857. doi: 10.1038/s41587-019-0209-9.

Martino, C., Morton, J. T., Marotz, C. A., Thompson, L. R., Tripathi, A., Knight, R., et al. (2019). A Novel Sparse Compositional Technique Reveals Microbial Perturbations. mSystems 4, e00016-19. doi: 10.1128/mSystems.00016-19.
